# Review of Programs for Persons Facing Death with Dementia

**DOI:** 10.3390/healthcare7020062

**Published:** 2019-04-15

**Authors:** Ladislav Volicer

**Affiliations:** 1 School of Aging Studies, University of South Florida, Tampa, FL 34639, USA; lvolicer@usf.edu; Tel.: +1-813-909-0539; 2 The 3rd Medical Faculty, Charles University, 11000 Prague, Czech Republic

**Keywords:** dementia, end-of-life, activities, quality of life

## Abstract

Background: Persons with advanced dementia cannot initiate activities because of the executive dysfunction. The lack of activities was identified as one of the most important factors contributing to behavioral problems of these persons. The unmet needs were boredom/sensory deprivation, loneliness/need for social interaction, and need for meaningful activities. There is a need for activities designed specifically for residents with advanced dementia. Objective: A description of patient’s needs and of programs that intend to maintain quality of life for people with dementia and facing death. Data sources: A literature review of programs used for persons with advanced dementia and residing in long-term facilities, using the PubMed data base and collateral sources. Results: Since palliative care is appropriate for persons with advanced dementia, attention has to be paid to three following important aspects of care: Medical issues, behavioral symptoms, and meaningful activities. Medical interventions should be limited to those which have more benefits than burdens, behavioral symptoms should be distinguished according to the context in which they occur, and treated by non-pharmacological interventions that involve meaningful activities. This review describes four programs that may promote the quality of life in persons with advanced dementia and facing death. They are designed for persons with advanced dementia, taking into account their functional impairments. Most of these programs involve short infrequent sessions. In contrast, Namaste Care is a daily extended program of enhanced nursing care that can provide quality of life until the last breath. Conclusions: It is possible to maintain quality life for people with advanced dementia if a special program of activities is available.

## 1. Introduction

**Figure 1:**
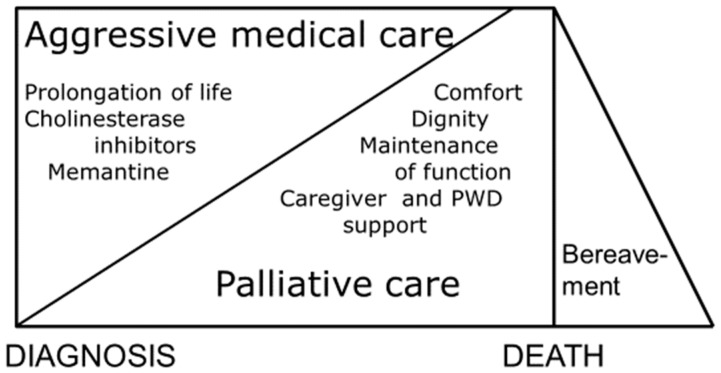
Types of dementia care (PWD = person with dementia) (reprinted with permission from Progress in Palliative Care 21(3), 146–150, 2013) [3].

With people living longer, an increasing number of them will die with some cognitive impairment, most likely Alzheimer’s disease. It is important that individuals who are starting to develop cognitive difficulties and mild cognitive impairment recognize the terminal nature of Alzheimer’s disease and other progressive degenerative dementias. However, there is a difference between terminal cancer and Alzheimer’s disease. Alzheimer’s disease does not cause death by itself. People with advanced Alzheimer’s disease die of a complication, most likely pneumonia [1]. Alzheimer’s disease is terminal in a different way than cancer. Soon after its onset, Alzheimer’s disease terminates the ability to remember new information and terminates the ability to initiate independent activities. Later on, Alzheimer’s disease terminates the ability to be independent in the activity of daily living and the ability to communicate verbally. Finally, if the person lives long enough, Alzheimer’s disease terminates the ability to walk, eat, and drink independently. As a consequence of the terminal nature of Alzheimer’s disease, a palliative approach to care is indicated [2]. However, the palliative approach does not eliminate aggressive medical care that may be provided simultaneously. The importance of these two types of care changes during the course of dementia, as is depicted in Figure 1.

When families and persons with dementia recognize the terminal nature of Alzheimer’s disease they need to prepare for this inexorable future. Although we hope that an effective treatment will be developed in the future, the probability of that is quite low. The current theory that Alzheimer’s disease is caused by accumulation of beta amyloid deposits in the brain may be incorrect [4]. Some pharmaceutical companies have stopped their effort to develop effective medications for the treatment of Alzheimer’s disease [5]. It is, however, possible to maintain the quality of life for people with Alzheimer’s disease and other progressive dementias, despite the terminal nature described above. To achieve this, it is necessary to address the three following main aspects of care: Medical issues, behavioral symptoms, and provision of meaningful activities. 

### 1.1. Medical Issues

Persons with dementia often have other comorbidities because dementia does not protect against other diseases [6]. Treatment of these comorbidities should take into consideration in the presence of dementia. Persons with dementia may be unable to report symptoms of diseases and also symptoms caused by the treatment. Therefore, it is important to avoid efforts to maintain normal blood pressure in hypertension that may lead to dizziness and falls and efforts to maintain low glycemia in diabetes that can cause hypoglycemia. Since dementia decreases life expectancy, it may be inappropriate to provide treatment with long-term effects, e.g., use of statins to treat hypercholesterolemia [7]. Prior to the initiation of a treatment, its costs and benefits should be considered. Any therapeutic intervention may cause discomfort for persons with dementia, especially if they do not understand the need for it. To ensure ethical care, the choice of treatments should be guided by the person’s goals of care [8].

**Figure 2:**
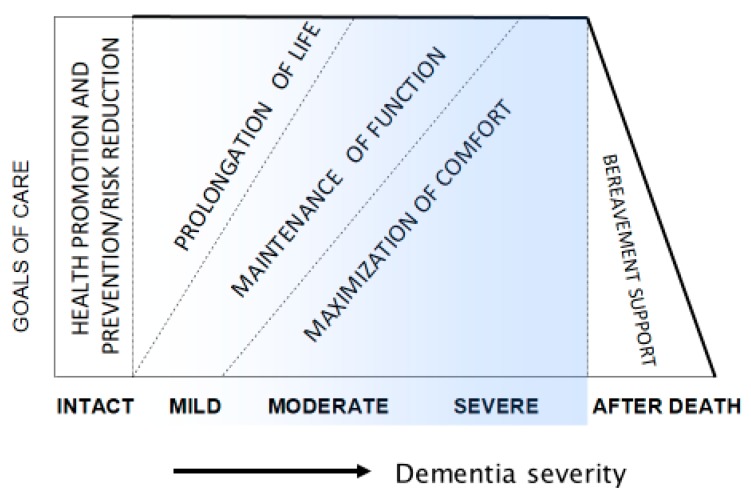
Dementia progression and prioritizing of care goals (reprinted with permission from van der Steen et al., Palliative Medicine 28(3), 197–209, 2014) [2].

There are three possible goals of care, as follows: Life prolongation, maintenance of functions, and maximal comfort [8]. It is not possible to meet all these goals at the same time, because prolongation of life may require aggressive medical interventions and hospitalization, which cause discomfort and loss of function. Therefore, it is important to prioritize the goals of care and to guide the care according to person’s priorities, which should be considered in advance directives. The priorities may change during the course of dementia, with life prolongation most important early on and assurance of comfort most important in the advanced and terminal stages (Figure 2).

Assurance of comfort can be promoted by avoiding some aggressive medical interventions that inflict more burden than benefits. These interventions include cardiopulmonary resuscitation, transfer to an acute care setting, tube feeding, and using antibiotics to treat generalized infections. However, advance directives which omit these interventions do not assure that the person will not live with advanced dementia because these interventions may not be needed. There may be no cardiac arrest and need to transfer to an acute care setting, people can a live long time with hand feeding and drinking with assistance, and pneumonia may resolve without antibiotics, especially if the person is well hydrated [9]. 

For those who fear living with advanced dementia and want to avoid it at all cost, the only effective way is to stop eating and drinking. This leads to death from dehydration, which was considered better than death from physician assisted suicide [10]. The person could specify in advance directives that at certain stage of dementia, when he/she can no longer eat and drink without assistance, he/she does not want to be helped with eating and drinking. Such an advance directive was acceptable to most relatives whose loved one died from dementia [11] and would be honored by most health care professionals [12].

### 1.2. Behavioral Symptoms

It is important to differentiate the two following types of behavioral symptoms: Symptoms that occur when the person with dementia is solitary and symptoms that occur when person with dementia is interacting with others. The most common solitary symptoms are apathy and agitation. The most common symptoms occurring when interacting with others is rejection of care that can escalate into reactive aggression. Unfortunately, these two types are sometimes not differentiated and all behavioral symptoms of dementia are called agitation [13] or behavioral and psychological symptoms of dementia (BPSD), ignoring that different symptoms may require different management strategy. 

#### 1.2.1. Apathy

Apathy is a very common symptom in people with dementia and sometimes may be the first symptom occurring during dementia development [14]. Apathy is different from depression and has specific diagnostic criteria [15]. Since persons who are apathetic do not represent any problems for their care providers, apathy is often not recognized and treated. Untreated apathy decreases the quality of life for persons with dementia [16] and may cause weight loss [17]. The severity of apathy increases with the severity of dementia and apathy is present in up to 92% of persons with advanced dementia [18]. Apathy was reported to be associated with increased caregiver burden [19], reduced independence in activities of daily living, and poor results in rehabilitation [20]. There is no medication that is approved specifically for the treatment of apathy and the most effective management are non-pharmacological strategies that provide meaningful activities which will be described in a later section. 

#### 1.2.2. Agitation

Agitation could be defined as “motor restlessness, heightened responsivity to stimuli, irritability, inappropriate and/or purposeless verbal of motor activity, decreased sleep and fluctuation of symptoms over time” [21]. Agitation may have already occurred in persons with mild cognitive impairment and the prevalence is similar in different stages of dementia. In contrast, rejection of care and possible reactive aggression start occurring only in moderate dementia and the prevalence increases with dementia progression [22]. Separation of agitation and aggression is also present in many scales used for measuring behavioral symptoms of dementia [23].

Agitation may be elicited by physical conditions and environmental factors. Physical conditions may include pain, hunger, thirst, decompensated chronic conditions (e.g., congestive heart failure and chronic obstructive pulmonary disease), organ failure, dehydration, and acid-base imbalance. Environmental factors include restraints, exit control, noise, and uncomfortable temperatures. Another risk factor for developing agitation is depression. Depressive symptoms are more common in persons with agitation than in persons without it, and when the severity of agitation changes, the number of depressive symptoms change in the same direction [24]. One of the most important factors causing development of agitation is lack of meaningful activities, which causes boredom. Thus provision of meaningful activities may be the most effective strategy for preventing and treating agitation [25].

#### 1.2.3. Aggression

Persons with moderate and severe dementia develop aphasia, which makes communication between them and their care providers difficult. Therefore, they may not understand the intentions of their care providers and need for the care. They may not cooperate with the care providers and reject the care. If the care providers insist on providing the care, persons with dementia may defend themselves, become combative, and develop reactive aggression. Labeling persons with dementia “aggressive” is really blaming the victim because persons with dementia in this situation consider the care provider as the aggressor. Another type of aggression, proactive aggression, is very rare because it requires planning and premeditation and this may not be possible to do for persons with dementia, because of the impairment of executive function. 

A lack of understanding leading to rejection of care is the most important factor for development of reactive aggression. The second most important factor leading to rejection of care is depression, with hallucinations and delusions having a minor role [26]. Thus, the most effective strategy for prevention and treatment of agitation is treatment of depression and an improvement in communication between persons with dementia and their care providers. Depression may actually lead to verbal abuse, even in persons with dementia who do not reject care. Results of some studies question the effectiveness of antidepressants in the treatment of behavioral symptoms of dementia. This may be caused by the ineffective treatment of depression. Results of the Depression in Alzheimer’s Disease Study clearly showed that the behavioral symptoms improve if depression is controlled, while they remain if the depression treatment is ineffective [27].

Communication with persons with dementia may be improved by training of the care providers [28] and by using cognitive-linguistic stimulation [29]. Postponing the care activity until the person with dementia is more agreeable and changing caregiving strategies are also important for decreasing the rejection of care [30]. Nonverbal communication could be improved by massage therapy [31], which makes persons with dementia used to touch. Therefore, they may not reject this care.

### 1.3. Meaningful Activities

Many programs were developed to improve psychosocial outcomes in persons with dementia [32], but most of them require that participants have verbal or motor abilities that persons with advanced dementia lack. Therefore, persons with advanced dementia either do not attend the activities and are isolated in their rooms or hallways or sit on the periphery of an activity, not participating and mostly sleeping [33]. Therefore, they need programs specifically designed for persons with advanced dementia that recognize their functional limitations. A literature review found descriptions of four such programs and they are described below.

## 2. Materials and Methods

**Figure 3:**
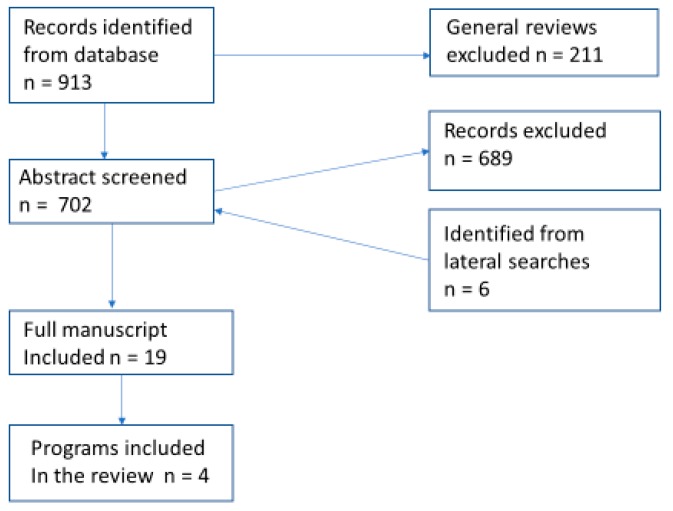
Overview of the study selection process.

A search was conducted by opening the PubMed program and searching with keywords “advanced dementia AND activities”. All articles published until the beginning of 2018 and all languages were considered. This search yielded 913 publications (Figure 3). General review articles were excluded and the remaining 702 abstracts were screened. The criteria for the inclusion of articles in the review included advanced stages of dementia of the participants in described program, non-pharmacological intervention, and implementation of the program in a long-term care setting. If the abstract seemed to be acceptable, the whole publication was reviewed. This screening excluded 689 publications, but 6 publications were added based on further review of literature and reviewers’ recommendations. Both scholarly and non-scholarly literature were used. This selection resulted in 19 publications describing 4 different programs. None of these publications had any ethical issues.

## 3. Results

There are four programs that can be used for persons with advanced dementia, as follows: Snoezelen therapy, multisensory and motor-based group activity programs, Garden Experience, and Namaste Care. The articles that were found for individual programs are listed in Table 1.

### 3.1. Snoezelen Therapy

This program is also called the multisensory environment (MSE) [34] and the multisensory stimulation environment (MSSE) [35]. It was initially developed for people with severe learning disabilities, but it was also used for more than 20 years for people with dementia. This program incorporates equipment that is designed to stimulate the senses of sight, sound, touch, smell, and movement. The equipment includes bubble tubes that change color and speed in response to sound, swing chairs with colorful wall murals around it, mobiles of fish, mirrored balls, aroma diffusers, music systems, and projectors with colored light wheels that rotate slowly, providing gently changing colors and designs. The space used for the equipment is varied, from several rooms [36], to portable equipment using just a Snoezelen projector, and a stereo system for relaxing music [37]. This equipment is usually installed in a specific room with white walls into which persons with dementia are brought. 

The effectiveness of Snoezelen therapy is questionable. Although there are some positive results reported [35,37], a more recent study did not find any significant changes in agitation after Snoezelen use [38]. An ethnographic study of Snoezelen use found that equipment predominantly stimulates vision and touch, equipment is underused, and staff receive little training in how to facilitate sessions [34]. Snoezelen therapy may be less effective because it is limited by significant staff involvement and involves usually only short sessions, sometimes once a week. It is important to avoid sudden flashes in the Snoezelen room, because some persons may be bothered by reflections from the mirror ball as it turns. Some persons with advanced dementia may also find it confusing if they transferred from a familiar environment to the Snoezelen room. Other problems with Snoezelen are that some people might find the environment very artificial and that the equipment is quite expensive [39].

### 3.2. Multisensory and Motor Based Group Activity Program

In contrast to Snoezelen, this program stimulates all senses, including the gustatory sense, by using natural items. Presentation of the senses is arranged in a specific order, starting with smell and gross motor activity to evoke general arousal and alertness. Touch, visual, and auditory cues provide increasing complexity of stimuli and require higher-level interpretative skills. The taste experience is used at the end of the group session because of its rewarding and reinforcing effect and it encourages informal socialization. All sensory cues are selected to be pleasurable and novel [40].

The program was first developed for group activities [40] but later was applied to improve the bathing experience of persons with advanced dementia [41]. To implement this program the staff participated in training sessions, devoted to stimulation of one of the following senses: Olfaction, communication/contact, vision, audition, and taste. Additional sessions addressed residents’ engagement, challenging behaviors, and safe environment. This program resulted in the significant increase of bathing time in which the residents were engaged, increased caregiver-direct gaze and laughing, and decreased duration of closed eyes [42]. Unfortunately, a longitudinal study found the effects of training decreased with time [43], which is a problem with many training based programs. 

Group activities using this approach consist of thematic sessions in which all senses are stimulated by objects related to the theme (Table 2). The original study consisted of three 1-hour sensory stimulation treatment sessions per week for 6 weeks and included 6 subjects in a control group and 7 subjects in an experimental group. The study did not find any statistically significant differences in functional performance between the control and experimental groups, but qualitative data indicated that all the subjects tolerated the sessions and were engaged with the stimuli [40]. Another study, providing 16 weekly sessions to 4 residents with moderate to severe dementia, found that one participant was not engaged and the others showed variable levels of engagement [44]. Thus, the effects of this activity program on quality of life need further investigations.

### 3.3. Garden Experience

There is some evidence that being in a garden or viewing a garden could be beneficial for persons with advanced dementia. A qualitative study reported that adapted gardening was a constructive outdoor activity for persons with advanced Huntington’s disease [45], which promoted social interaction and physical activity. Some staff used the garden for therapy and visitors used the garden to meet with the residents socially.

Viewing a Japanese garden for 15 min twice a week was reported to induce positive behavioral changes and a decreased heart rate, indicating a relaxation state. By contrast, in the Snoezelen room heart rate did not decrease and responses of the subjects were more negative. When the Japanese garden was replaced by furniture, subjects had negative behavioral signs after being returned to the same room [46]. Further research, using a Japanese garden on a roof, found that the subjects scanned a larger area when viewing the garden and again had a lower heart rate and improved behavioral symptoms [47].

The problem with garden experience is that it is influenced by weather. When the garden was closed by sliding glass doors because of inclement weather, viewing the garden though the glass doors did not have a beneficial effect, even when a chrysanthemum scent was added [47]. 

### 3.4. Namaste Care

**Figure 4:**
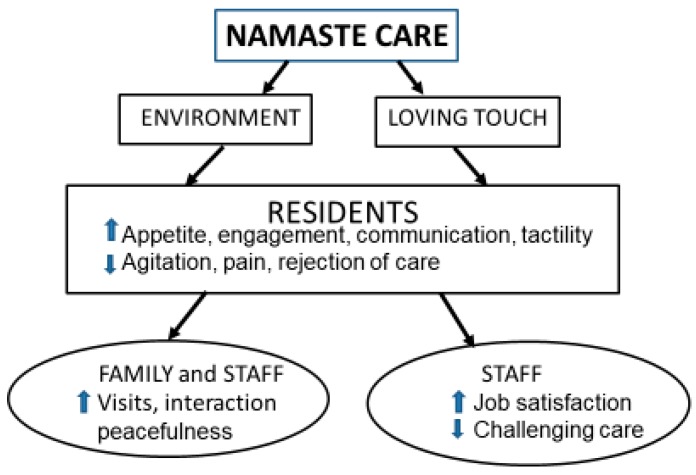
Namaste Care principles and consequences.

Namaste is an Indian greeting, which means “I honor the spirit within you”, and was used as a name for this program because its goal is to honor the spirit in persons with advanced dementia. Namaste Care has two main principles, creating a comfortable environment with the presence of others and a loving touch approach to all activities [48] (Figure 4). The program was originally developed for persons with advanced dementia who reside in a nursing home or an assisted living facility. However, it was found useful for persons with other diagnoses who do not benefit from traditional activities [49]. In contrast to the two programs described above, Namaste Care does not consist of short programs few days a week, it provides activity for 4 h a day (2 h in the morning and 2 h in the afternoon), when possible, for 7 days a week. It is an enhanced nursing program which does not require highly qualified activity professionals or additional staff. It is usually run by an existing staff member who cares for the usual number of patients in a Namaste room (6–8), some of them assigned to her while other staff members care for patients who were assigned to her and are not in the Namaste room. Namaste Care does not require additional staff and expensive equipment, except for comfortable seating. 

The Namaste room does not have to be a dedicated space, it can be multiservice place, e.g., family visiting room or part of a dining room, which is reserved for Namaste Care times. It should be as free from distractions as possible, lights are lowered, relaxing music is played, and an enjoyable scent, e.g., lavender, permeates the room. The person leading the program for the day prepares the room and gathers all supplies needed for the program so the residents are never left alone. Residents who participate in Namaste Care are brought in by other staff, which may even include personnel. Each resident is greeted in a personalized manner, some with the hand shake other with the hug. If the residents are not in a reclining chair, they are placed in one because wheel chair is not comfortable seating for persons with advanced dementia. They are made comfortable using pillows and their special blankets, assessed for pain [54], and offered their favorite beverage. Beverages are offered continuously throughout Namaste Care sessions to improve hydration. All activities are meaningful to the resident, performed slowly, and offered with a loving touch approach. Morning activities include application of a familiar scented face moisturizer for the ladies, gently combing their hair, and gentle massage on the arms and hands. The men may enjoy a scalp massage and an old-fashioned shave using shaving cream, a safety razor, and an aftershave lotion that they may have used in the past. The person leading the Namaste Care program speaks to the residents during all interactions, even if the resident is non-verbal [55]. 

The Namaste Care program was originally designed as a group activity for nursing homes and assisted living facilities. However, it can be also provided on an individual basis in residents’ rooms or at home, e.g., for patients cared for by a hospice program. One national hospice organization recognized that the Namaste Care approach is so comforting that it is providing it for all patients, not just those with cognitive impairment. Namaste Care programs are currently offered in a variety of settings in 10 countries and receive good reception everywhere [50,51,56].

Namaste Care was found to improve the quality of life of nursing home residents with advanced dementia [48], decrease behavioral symptoms of dementia [52], and this allowed the discontinuation of antipsychotic medications [53]. Decreased rejection of care is probably mediated by the loving touch the residents receive during Namaste Care sessions, which makes them less likely to resist care activities involving touch even outside of the Namaste Care program. Since the Namaste Care program provides stimulation during the sessions, the residents are less likely to sleep during the day and there is less need for hypnotic medications [53]. There is some evidence that Namaste Care decreases depressive symptoms, improves the ability to communicate with family members and staff, and decreases complaints of pain. Namaste Care is well received by both family members of persons with dementia and by staff [48].

## 4. Discussion and Conclusions

Taking care of a person facing death with dementia requires attention to appropriate medical care, management of behavioral symptoms of dementia, and involvement in an appropriate activity program. Medical care should eliminate interventions which cause more burden than benefits and behavioral symptoms require treatment to prevent stressful situations for the person with dementia. 

This review found four programs that could be used for persons with advanced dementia. Three of the programs provide activities for a limited amount of time, once or twice a week. Although there is good evidence that persons with dementia are more relaxed and less agitated during the programs’ session, there is no evidence that the programs have a lasting effect. Namaste Care provides activities for several hours each day and results in lasting effects. This is especially due to frequent massage provided with loving touch, which increases the tolerance for touch during needed care. That decreases the tendency to reject care and eliminates combative behavior which may result in reactive aggression. 

It should be recognized that this review has some limitations. We might have missed some programs that are useful for persons with advanced dementia but were not described in journals included in the PubMed. We also eliminated programs that are used mainly during the process of dying, such as No One Dies Alone [57] and use of music therapy in hospice care [58]. The programs included in this review could be used during the last months or years, during which the person with dementia is unable to participate in activities suitable for people with less advanced dementia. The use of these programs allows people dying with dementia to live quality in their lives until they take their last breath.

## Figures and Tables

**Table 1:** Articles included in this literary review (for further description of the articles see text).

Programs	References	Intervention	Population	Conclusions
**Snoezelen therapy**	Collier, L.; Jakob, A. [34]	Variable use of equipment	16 care homes	Stimulation of vision and touch
	Sancheza, A.; et al. [35]	Two 30-min weekly sessions over 16 weeks	22 persons with severe dementia in two groups	Significant improvement in their RAID and BANS-S scores
	Cunningham, C.C. et al. [35]	Description of equipment	Varied	Improvement of behaviors
	Brown, E.J. [37]	60 min before dental treatment	36 persons with advanced dementia	Decreased resistive behavior
	Berkheimer, S.D.; et al. [39]	Three 30 min sessions for 3 weeks	8 persons with advanced dementia	None significant change of agitation
**Multisensory and Motor Based Group Activity Program**	Trudeau, S.A. [40]	3 one-hour sessions/week for 6 weeks	13 persons with advanced dementia	More engagement
	Cruz, J. et al. [41]	Implementation during morning care	6 residents with moderate/severe dementia	Improvement in laughing and engagement
	Sposito, G. et al. [42]	Implementation during morning care	45 residents with moderate/severe dementia	More engagement, direct gaze, less sadness
	Marques, A.; et al. [43]	Implementation during morning care	6 residents with moderate/severe dementia	Less effect with time
	Cruz, J.; et al. [44]	16 45-min weekly sessions	4 residents with advanced dementia	Active involvement and engagement
**Garden experience**	Spring, J.A.; et al. [45]	General garden use	7 residents, 3 staff, 2 relatives	Residents and visitors enjoying garden
	Goto, S.; et al. [46]	Twice/week for 15 min	36 residents	Decreased heart rate
	Goto, S. et al. [47]	Visit twice/week for two weeks	25 residents with dementia	Reduced heart rate and improved behavioral symptoms
**Namaste Care**	Manzar, B.A.; Volicer, L. [48]	Daily two 2-hour sessions	9 residents with advanced dementia	Improved quality of life, decreased behavioral symptoms
	Simard, J.; Volicer, L. [49]	Daily two 2-hour sessions	86 residents with advanced dementia	Decreased delirium indicators, better social interaction
	Magee, M. et al. [50]	Daily two 2-hour sessions	9 residents with advanced dementia	
	McNiel, P.; Westphal, J. [51]	Daily two 2-hour sessions	14 staff member interviews, 16 residents	Six beneficial themes
	Stacpoole, M. et al. [52]	Daily two 2-hour sessions	30 residents with advanced dementia	Decreased behavioral symptoms and pain
	Fullarton, J.; Volicer, L. [53]	Daily two 2-hour sessions	9 residents with advanced dementia	Decreased use of antipsychotics and hypnotics

**Table 2:** Sample of Motor and Multisensory Care sessions (reprinted from Trudeau SA. Bright Eyes: a structured sensory-stimulation intervention. In: Volicer L, Bloom-Charette L, editors. Enhancing the Quality of Life in Advanced Dementia. Philadelphia: Taylor & Francis; 1999. p. 93–106) [40].

Sense	The Beach	Trains	Baseball	Fishing	Gardening
Olfactory	Coconut suntan lotion	Ground coffee	Fresh-cut grass	Sardines	Garden fresh tomato
Kinesthetic	Beachball toss	Balloon volley	Soft baseball toss	Casting with rod and reel	“Digging” with shovel
Tactile	Terrycloth towel	Conductor’s cap	Felt baseball hats	Fishing flies (feather and thread)	Potting soil and trowel
Visual	Photos from Hawaiian calendar	Black and white train photos	Photo of local ballpark	Calendar of trout flies	Seed catalogs
Auditory	Ocean-waves tape	“Atchinson Topeka and the Santa Fe”	“Take me out to the Ballgame”	Seagull sounds	Tape of crickets at dusk
Gustatory	Cold lemonade	Chocolate cookies	Nonalcoholic beer	Sardines on saltine crackers	Peeled tomato
